# Chemistry in flow systems III

**DOI:** 10.3762/bjoc.9.193

**Published:** 2013-08-16

**Authors:** Andreas Kirschning

**Affiliations:** 1 Leibniz University of Hannover, Schneiderberg 1B, 30167 Hannover, Germany

**Keywords:** flow chemistry

The third Thematic Series on flow chemistry published in the Beilstein Journal of Organic Chemistry demonstrates the emerging importance of transforming chemical synthesis in the laboratory from a classical batch approach to continuous processes by using micro- and miniaturized flow reactors. In the past two decades this technology has seen a dramatic increase of visibility. Considering an analyses of the accompanied developments in flow synthesis one has to acknowledge that the topic has shifted among different disciplines with a variety of intensities and focus.

In the late eighties and nineties, the idea of miniaturising continuous chemical processes was mainly pursued by chemical engineers. They were fully aware of the quest for an intensification of the process and the advantages associated with continuously operated chemical processes. The benefits of miniaturizing flow systems are evident when considering the excellent heat and mass transfer properties of these small technical devices. Chemical engineers developed beautifully designed reactors, mixers and interfaces for the online monitoring of continuous processes. The major inspiration came from the process and development units in the chemical industry, where continuously operated pilot plants already played a key role. Conceptually, highly modular microreaction systems developed by Ehrfeld Mikrosystem BTS and by CPC (Cellular Process Chemistry Systems) are marvelous examples of these engineered driven achievements.

In the late nineties, organic chemists from both industry and academia, which included our group, became involved in the use of microreactors and provided a myriad of synthetic examples. The combined work of experts from engineering and chemical synthesis was highly fruitful and is so until today. This combination of expertise has catalyzed the development of microreactor technology in the applied context of synthesis and production.

Chemical engineers depend on input from chemists and synthetic chemists will only advance the field of miniaturized flow synthesis if they are aware of the technical and engineering aspects. This includes the quest for developing analytical devices for online monitoring and feedback loops for optimising synthetic protocols.

Several of these aspects can be found in this third Thematic Series on flow chemistry. I am thankful to all my colleagues who contributed with their excellent research to this issue. The Beilstein Team is acknowledged for the handling of the manuscripts and referee reports in a very pleasant and professional manner.

Andreas Kirschning

Hannover, August 2013

